# In active acromegaly, IGF1 bioactivity is related to soluble Klotho levels and quality of life

**DOI:** 10.1530/EC-14-0028

**Published:** 2014-04-15

**Authors:** A J Varewijck, A J van der Lely, S J C M M Neggers, S W J Lamberts, L J Hofland, J A M J L Janssen

**Affiliations:** 1 Erasmus MC, Department of Internal Medicine Division of Endocrinology 's-Gravendijkwal 230, Room D-443, Rotterdam, 3015 CE The Netherlands

**Keywords:** IGF1 bioactivity, soluble Klotho, total IGF1, acromegaly, quality of life

## Abstract

The value of measuring IGF1 bioactivity in active acromegaly is unknown. Soluble Klotho (S-Klotho) level is elevated in active acromegaly and it has been suggested that S-Klotho can inhibit activation of the IGF1 receptor (IGF1R). A cross-sectional study was carried out in 15 patients with active acromegaly based on clinical presentation, unsuppressed GH during an oral glucose tolerance test, and elevated total IGF1 levels (>+2 s.d.). Total IGF1 was measured by immunoassay, IGF1 bioactivity by the IGF1R kinase receptor activation assay and S-Klotho by an ELISA. Quality of Life (QoL) was assessed by Acromegaly QoL (AcroQoL) Questionnaire and Short-Form-36 Health Survey Questionnaire (SF-36). Out of 15 patients, nine had IGF1 bioactivity values within the reference range. S-Klotho was higher in active acromegaly compared with controls. Age-adjusted S-Klotho was significantly related to IGF1 bioactivity (*r*=0.75, *P*=0.002) and to total IGF1 (*r*=0.62, *P*=0.02). IGF1 bioactivity and total IGF1 were inversely related to the physical component summary of the SF-36 (*r*=−0.78, *P*=0.002 vs *r*=−0.60, *P*=0.03). Moreover, IGF1 bioactivity, but not total IGF1, was significantly inversely related to the physical dimension of the AcroQoL Questionnaire (*r*=−0.60, *P*=0.02 vs *r*=−0.37, *P*=0.19). In contrast to total IGF1, IGF1 bioactivity was within the reference range in a considerable number of subjects with active acromegaly. Elevated S-Klotho levels may have reduced IGF1 bioactivity. Moreover, IGF1 bioactivity was more strongly related to physical measures of QoL than total IGF1, suggesting that IGF1 bioactivity may better reflect physical limitations perceived in active acromegaly.

## Introduction

Acromegaly is characterized by excess secretion of growth hormone (GH) causing multisystem-associated morbidities and increased mortality. GH is considered as the main regulator of circulating total insulin-like growth factor 1 (IGF1). Total IGF1 is therefore routinely used for diagnosis and monitoring treatment of GH deficiency and acromegaly. Nevertheless, discrepancies between clinical findings, GH, and total IGF1 levels are frequently encountered in clinical practice.

Many of the methods used for the measurement of circulating total IGF1 may be hampered by the interferences of insulin-like growth factor (IGF)-binding proteins (IGFBPs) remaining after extraction (1). The most important reason why immunoreactive total IGF1 levels are still used to assess IGF1 bioactivity has been the lack of reliable assays to measure IGF1 bioactivity. An IGF1 receptor (IGF1R)-specific kinase receptor activation (KIRA) assay has been developed as an alternative method to evaluate circulating bioavailable IGF1 (2). The principle of the IGF1R KIRA assay is based on quantification of serum-induced IGF1R phosphorylation in cells transfected with the human *IGF1R* (2). Compared with current IGF1 immunoassays, this assay theoretically has the advantage of measuring the net effects of serum on IGF1R activation, as it does not ignore the modifying effects of IGFBPs, IGFBP proteases, and other factors that interact between IGFs and the IGF1R (3, 4). One of these factors might be soluble Klotho (S-Klotho). S-Klotho is a protein that has been reported to inhibit IGF1R (and insulin receptor) signaling by inhibiting tyrosine phosphorylation of both receptors and their downstream signaling proteins (i.e. IRS) (5, 6, 7).

Acromegaly is characterized by excessively high GH and (immunoreactive) total IGF1 levels. Recent data suggest that S-Klotho level is also elevated in patients with active acromegaly and that S-Klotho level decreases toward normal following removal of the GH-producing pituitary adenoma (8, 9).

Previously, we found that determination of IGF1 bioactivity may offer advantages in the evaluation of adult GH deficiency compared with total IGF1 (10). In another study, we found that IGF1 bioactivity reflects different aspects of quality of life (QoL) than total IGF1 in GH-deficient patients during GH treatment (11).

The aim of the present study was to investigate the value of IGF1 bioactivity in the evaluation of active acromegaly and in the assessment of QoL in active acromegaly. In addition, we studied whether S-Klotho levels are elevated in active acromegaly and whether a relationship exists between S-Klotho levels and IGF1.

## Subjects and methods

### Study population

This was an investigator-initiated cross-sectional study. A total of 15 patients, with active acromegaly, were enrolled. Diagnosis of active acromegaly was based on clinical presentation, unsuppressed GH levels during an oral glucose tolerance test (OGTT), and elevated age-matched total IGF1 levels (and radiological detection of a pituitary tumor). Of them, ten patients had a macroadenoma of the pituitary gland and five had a microadenoma. Body weight, height, blood pressure, and ring size were measured. BMI was calculated. The following laboratory assessments were conducted in the fasting state; total IGF1, IGF1 bioactivity, IGFBP1, IGFBP3, glucose, insulin, and S-Klotho. Three questionnaires were used to assess symptoms and QoL (see below).

All 15 patients had no previous history of surgery or radiotherapy for acromegaly. Informed consent was obtained from all subjects and the study was approved by the medical Ethics Committee of Erasmus MC.

### Blood measurements

Serum total IGF1, IGFBP3, GH, and insulin were initially measured by solid-phase, enzyme-labeled chemiluminescent immunometric assays (intra-assay coefficients of variation (CV) values were 3.9, 4.4, 3.5, and 3.3–5.5% and inter-assay CV values were 7.7, 6.6, 6.5, and 4.1–7.3% respectively; Immulite 2000 supplied by Siemens Medical Solutions Diagnostics, Los Angeles, CA, USA). Serum IGFBP1 was measured by the ELISA (intra-assay CV value was 7.4% and inter-assay CV value was 6.8%) (Mediagnost GmBH, Reutlingen, Germany). Serum glucose was determined with a standard laboratory method. S-Klotho was determined using a sandwich ELISA described by Yamazaki *et al*. (12) (Kyowa Hakko Kirin Co. Ltd, Tokyo, Japan) according to the manufacturer's instructions. As control group we measured S-Klotho in 11 subjects with hypopituitarism, who were receiving complete hormone replacement therapies for all present pituitary deficiencies. These subjects appeared to have S-Klotho concentrations that were comparable with those found previously in a healthy population (12).

IGF1 bioactivity was determined by the IGF1R KIRA assay. The IGF1R KIRA assay has been described previously (2, 3). Briefly, the IGF1R KIRA assay uses a HEK cell line that is stably transfected with the human *IGF1R* gene and quantifies phosphorylation of tyrosine residues of the transfected IGF1R to assess IGF1R stimulating activity. All measurements were done in duplicate. The intra- and inter-assay CV values were <15%.

Total IGF1 concentrations, IGF1 bioactivity, and IGFBP3 were compared with the age-specific normative range values that have been published previously (3, 13). Total IGF1, IGFBP3, and IGF1 bioactivity individual *Z*-scores were calculated using the following formula: *Z*-score=(*x*−average *x*/s.d.), where *x* is the actual total IGF1 level or IGFBP3 or IGF1 bioactivity, average *x* is the mean total IGF1 level or IGFBP3 or IGF1 bioactivity at that age, and s.d. is for the mean at that age. The percentage IGF1 bioactivity of total IGF1 was calculated using the formula: IGF1 bioactivity (pmol/l)/total IGF1 (pmol/l)×100%.

### QoL Questionnaires

#### Acromegaly Quality of Life Questionnaire

The Acromegaly Quality of Life (AcroQoL) Questionnaire comprises 22 questions. Each question has five possible answers scored 1–5, with a total maximum score of 110 and quoted as a percentage. The score of 110 reflects the best possible global AcroQoL score. The 22 questions are divided into two main categories: physical and psychological function. The psychological dimension is subdivided into appearance and personal relationships (14, 15). The AcroQoL Questionnaire has a good internal consistency (Cronbach's *α*>0.7) (16).

#### Short-Form-36 Health Survey Questionnaire

The Short-Form-36 Health Survey Questionnaire (SF-36) is a widely used generic measure of health status (17). The mental and physical component summaries (MCS and PCS respectively) of the SF-36 were calculated by standardizing the subscale scores using a linear *Z*-score transformation using national (Dutch) means and s.d. Then, *Z*-scores were multiplied by the US subscale factor score coefficients for PCS and MCS and summed over all eight subscales. Finally, *T*-scores were calculated by multiplying the obtained PCS and MCS sums by ten and adding 50 to the product, to yield a mean of 50 and a s.d. of ten for the norm population (18).

### Patient-Assessed Acromegaly Symptom Questionnaire

The Patient-Assessed Acromegaly Symptom Questionnaire (PASQ) assesses symptoms and is a disease-specific questionnaire. It consists of six questions scoring 0–8 and the seventh question addressing the overall health status, based on the other six questions, scoring 0–10 (19). The first six questions evaluate symptoms such as headache, excessive sweating, joint pain, fatigue, soft tissue swelling, and numbness or tingling of the extremities. The maximum score of these six questions is 48 and indicates severe symptoms, with lower scores reflecting milder symptoms.

### Statistical analysis

The clinical characteristics of the study population are presented as means with s.d. or ranges. The Kolmogorov–Smirnov test was used to test normality of the variables (data were considered to be normally distributed when *P*>0.05). For data that do not meet the criteria for normality, logarithmic transformations were applied. Age-adjusted Pearson's correlations were calculated to assess associations. Differences between groups were calculated by the Wilcoxon signed-rank test.

A *P* value of 0.05 or less was considered statistically significant. Data were analyzed using SPSS 20 for Windows (SPSS, Inc.).

## Results

### Clinical characteristics of study population

Table 1 lists the clinical characteristics of the study population. Three patients received metformin therapy for type 2 diabetes mellitus. Two of these three patients were treated with insulin and six patients were on antihypertensive treatment. One patient had abnormal renal function and one patient had a history of laryngeal cancer *in situ*. None of the subjects had abnormal liver functions.

Mean (median±s.d.) total IGF1 was 79.7 nmol/l (82.6±37.2) and the mean (median±s.d.) *Z*-score was 10.9 (11.8±6.4). Table 2 lists individual total IGF1 levels, IGF1 bioactivity, and *Z*-scores of all 15 untreated subjects with active acromegaly. All total IGF1 concentrations were above+2 s.d., which was a prerequisite criterion for the inclusion in this study (Table 2). Mean (median±s.d.) IGF1 bioactivity was 589 pmol/l (545±237); mean (median±s.d.) *Z*-score for IGF1 bioactivity was 1.77 (1.29±2.15); nine out of 15 patients (60%) had IGF1 bioactivity values within the reference range (Table 2). After excluding the diabetic subjects from the analysis, mean (median±s.d.) total IGF1 was 75.9 nmol/l (76.3±7.1) and the mean (median±s.d.) *Z*-score was 10.5 (10.7±7.1), while mean (median±s.d.) IGF1 bioactivity was 632 pmol/l (641±243) and the mean (median±s.d.) *Z*-score was 2.24 (2.19±2.13). The percentage IGF1 bioactivity (mean (median±s.d.) of total IGF1 was 0.81 (0.74±0.30). Mean (median±s.d.) IGFBP3 concentration was 7.0 mg/l (7.5±1.5) mg/l, mean (median±s.d.) *Z*-score for IGFBP3 was 2.58 (2.95±1.46) s.d. Out of 15 patients, eight had values for IGFBP3 above+2 s.d.

**Figure 1:**
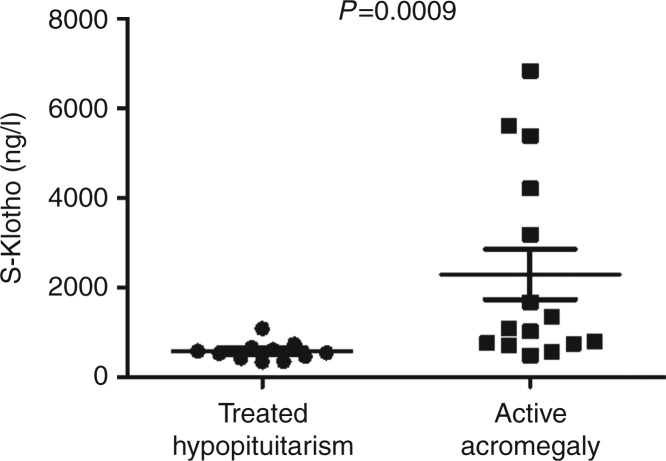
Mean (±s.e.m.) S-Klotho levels in subjects with active acromegaly (right) and in subjects with hypopituitarism, who were receiving complete hormone replacement therapies for all present pituitary deficiencies (left) (see text for details).

Serum S-Klotho levels in subjects with active acromegaly were significantly higher than that in subjects with hypopituitarism, who were receiving complete hormone replacement therapies for all pituitary deficits (*n*=11): 2291 ng/l (488–6823) (mean (range)) vs 574 ng/l (341–1084); *P*=0.0009 (Fig. 1). Thus, in our study, subjects with hypopituitarism had comparable S-Klotho levels as was reported previously in a healthy population (12).

### Age-adjusted relationships between IGF1, IGFBPs, GH, and S-Klotho

Total IGF1 was positively related to IGF1 bioactivity (*r*=0.86, *P*<0.001; *R* ^2^=0.70) and IGFBP3 (*r*=0.64, *P*=0.02), while the relationships between total IGF1 and random GH (*r*=0.52, *P*=0.07) and between total IGF1 and IGFBP1 (*r*=−0.55, *P*=0.05) just missed statistical significance. IGF1 bioactivity was positively related to IGFBP3 (*r*=0.59, *P*=0.03) and random GH (*r*=0.63, *P*=0.02) and negatively to IGFBP1 (*r*=−0.58, *P*=0.04).

**Figure 2:**
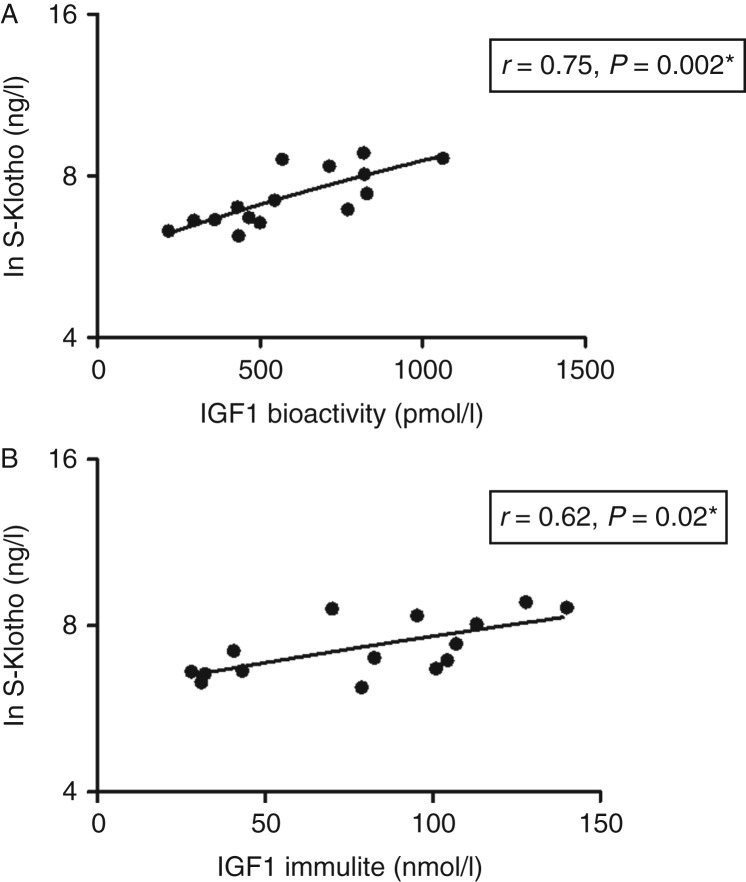
Relationships between the natural logarithm of serum S-Klotho (Ln S-Klotho) levels and IGF1 bioactivity (A) and total IGF1 levels (B) in active acromegaly. *Age-adjusted.

S-Klotho was positively related to IGF1 bioactivity (*r*=0.75, *P*=0.002; Fig. 2A) and total IGF1 (*r*=0.62, *P*=0.02; Fig. 2B). There were no relationships between S-Klotho, IGFBP3 (*r*=0.40, *P*=0.18), IGFBP1 (*r*=−0.43, *P*=0.14), and random GH (*r*=0.37, *P*=0.21). In addition, the age-adjusted relationships between S-Klotho and insulin (*r*=0.48, *P*=0.08) and between S-Klotho and fasting glucose levels (*r*=0.55, *P*=0.08) just missed statistical significance.

### QoL measurements and PASQ

The mean (s.d.) and ranges of the PCS of the SF-36, the MCS of the SF-36, the AcroQoL Questionnaire, and the PASQ score in 15 patients with active acromegaly are given in Table 3. The age-adjusted relationships between IGF1 parameters, measures of QoL, and the PASQ are given in Table 4. Both total IGF1 and IGF1 bioactivity were inversely related to the PCS of the SF-36 and were positively related to PASQ. Moreover, IGF1 bioactivity, but not total IGF1, was significantly inversely related to the physical dimension of the AcroQoL Questionnaire. S-Klotho was not related to any of the QoL measurements (data not shown).

## Discussion

In this cross-sectional study of newly diagnosed patients with active acromegaly based on clinical presentation, unsuppressed GH levels during an OGTT, and elevated age-matched immunoreactive total IGF1 levels, IGF1 bioactivity was within the reference range in a considerable number of patients.

This result suggests that, in subjects with active acromegaly, total IGF1 level is more often elevated than IGF1 bioactivity as all subjects had total IGF1 outside the reference range. However, clinical presentation with elevated age-matched total IGF1 levels was a prerequisite criterion for the inclusion in this study and this may have introduced a selection bias.

Most recent studies have consistently found that IGF1 levels are lower in diabetic patients than in healthy volunteers (20). In our study, three subjects received metformin for type 2 diabetes mellitus and two were additionally treated with insulin. Excluding diabetic subjects from the analysis did not change the mean results for total IGF1. However, the mean IGF1 bioactivity became >+2 s.d. suggesting that diabetes can reduce IGF1 bioactivity in acromegalic subjects.

Previously, we have shown in a healthy population that 70% of the variation in IGF1 bioactivity could not be explained by levels of total IGF1 (3). In this study, the *R* ^2^ value was 0.70 suggesting that 30% of the variation in IGF1 bioactivity could not be explained by levels of total IGF1, again demonstrating that IGF1 bioactivity is only partly dependent on total IGF1. In addition, the mean percentage of IGF1 bioactivity over total IGF1 was 0.81% in the 15 subjects with active acromegaly indicating that the IGF1 KIRA assay provides information about the circulating IGF1 system that fundamentally differs from that obtained by IGF immunoassays.

IGF1 bioactivity was within the reference range in a considerable number of subjects. However, this finding does not mean that IGF1 bioactivity was normal for these subjects. The overall variation in the healthy population determines the width of the population-based reference range for IGF1, which is defined by a distribution that includes 95% of the test results in a healthy population (21). As a consequence, any pathological change in IGF1 in a (acromegalic) subject must be very large (id est outside the boundaries of the population-based reference range) to be clinically considered as abnormal. However, the dispersion of ‘normal’ IGF1 in a healthy individual will usually span only a small part of the conventional population-based reference range (22). Therefore, an IGF1 bioactivity value within the reference range may be still abnormal for an individual (and provides arguments for developing specific individual reference range values for IGF1 (bioactivity) in this era of personalized medicine).

Interestingly, our finding that IGF1 bioactivity was within the reference range in a considerable number of patients with active acromegaly is in agreement with the results published in the 1970s and 1980s, which were obtained using classical IGF1 bioassays based on cartilage stimulation (23, 24). In those days, it was already postulated that discrepancies between IGF1 bioassays and total IGF1 immunoassays were reflecting effects of somatomedin inhibitors and other biologically relevant factors that were apparently detected by bioassays but recognized poorly by immunoassays (23).

Recent data suggest that S-Klotho level is dramatically elevated in patients with active acromegaly and decreases toward normal following removal of the GH-producing pituitary adenoma (8, 9). As levels of S-Klotho are markedly elevated in relation to GH excess in acromegaly, it has been suggested that S-Klotho levels depend on GH to a comparable extent as IGF1 (7). In our study, we found elevated S-Klotho levels in active acromegaly. In addition, we observed a significant positive relationship between S-Klotho and IGF1. There are at least two potential explanations for these positive relationships between S-Klotho and IGF1 in our study. First, S-Klotho and IGF1 may both be independent markers for the severity of acromegaly. Another possibility could be that the elevation in S-Klotho levels represents an adaptation mechanism by which the body attenuates increased IGF1 action. In favor of this latter possibility, it has been found that S-Klotho can inhibit the activation of the IGF1R in a dose-dependent manner (5, 25). It suppresses not only ligand-stimulated autophosphorylation of the IGF1R but also activation of signaling events downstream of the IGF1R (5, 26), which in turn may reduce the activation of the IGF1R in patients with active acromegaly. To the best of our knowledge, we are the first to present data suggesting that increased S-Klotho levels are part of a physiological mechanism dampening IGF1 actions in active acromegaly patients.

S-Klotho-induced IGF1R resistance may help to explain why diabetogenic effects of elevated GH levels in active acromegalic subjects are often not completely counteracted and neutralized by IGF1 (27, 28).

As discussed above, the IGF1 KIRA assay, in contrast to total IGF1 immunoassays, quantifies autophosphorylation of the IGF1R. This opens the possibility that the KIRA assay is sensitive to the modifying actions of circulating S-Klotho and this may have contributed to the observed discrepancy between IGF1 bioactivity and total IGF1 in our study.

It has been suggested that patients' perception of QoL cannot be inferred directly from circulating hormone levels (29). However, there was an inverse relationship between the PCS of the SF-36 and IGF1 bioactivity and total IGF1. This latter relationship was stronger for IGF1 bioactivity than for total IGF1. In addition, IGF1 bioactivity, but not total IGF1, was significantly related to the physical dimension of the disease-specific AcroQoL Questionnaire.

Our results suggest that IGF1 bioactivity may better reflect physical limitations perceived by untreated acromegalic patients than total IGF1. In this respect, it would be interesting to measure IGF1 bioactivity, total IGF1, and S-Klotho levels in a group of patients with acromegaly, in which total IGF1 levels remain high during treatment with somatostatin analogs despite improvement in clinical symptoms. Such a study may help to give more insight into the known lack of correlation between total IGF1 and clinical scores during medical therapy of some acromegalic patients.

Both IGF1 bioactivity and total IGF1 were not related to MCS. In this respect, it is important to emphasize that it was previously found that physical function dimensions of the SF-36 are perceived lower in subjects with acromegaly than in the general population, while no differences have been found in the mental dimensions of the SF-36 (30).

Finally, though the disease-specific PASQ still requires validation before it can be used in everyday clinical practice, both IGF1 bioactivity and total IGF1 were significantly related to the PASQ. However, this latter relationship was stronger for total IGF1 than for IGF1 bioactivity.

Although the IGF1 KIRA assay provides a reproducible method for quantitatively assessing IGF1 bioactivity, currently it does not have a clear place in clinical routine due to several limitations such as assay run time, its labor intensiveness, and expenses higher than IGF1 immunoassays. Taking this into consideration, an important step toward implementation of the IGF1 KIRA assay into clinical routine is to run the assays on an automated system so that the assay can be performed with minimal personnel involvement at each step from beginning to end.

It should be stressed that our study has a considerable number of limitations. First of all, only a small number of acromegalic patients were studied and it had a cross-sectional study design. Patients were initially selected on clinical presentation and elevated total IGF1 levels. Moreover, only fasting hormone levels were measured, and there was no information on 24 h GH secretion nor on IGFBP protease activity.

In conclusion, IGF1 bioactivity was within the reference range in a considerable number of patients with active acromegaly in contrast to total IGF1. As the IGF1 KIRA assay, unlike the IGF1 immunoassay, does not ignore the modifying effects of factors that interact between IGFs and the IGF1R, S-Klotho may have reduced IGF1 bioactivity. In addition, IGF1 bioactivity was also more strongly related to physical measures of QoL than total IGF1, suggesting that IGF1 bioactivity may better reflect physical limitations perceived in patients with active acromegaly.

Our study suggests that measuring IGF1 bioactivity in subjects with active acromegaly provides information about the IGF1 system, which fundamentally differs from that obtained by common available IGF immunoassays. Larger studies are needed to confirm our findings.

## Author contribution statement

A J Varewijck researched data and wrote the manuscript, A J van der Lely, S W J Lamberts, and L J Hofland reviewed/edited the manuscript and contributed to the discussion, S J C M M Neggers researched data, reviewed/edited the manuscript, and contributed to the discussion, and J A M J L Janssen researched data, wrote the manuscript, and reviewed/edited the manuscript.

## Figures and Tables

**Table 1:** Clinical characteristics of the study population (*n*=15; 12 males and three females).

**Clinical characteristics**	**Mean** (s.d.)	**Range**
Age (years)	52.8 (13.9)	24.1–69.7
BMI (kg/m^2^)	30.1 (6.2)	21.4–41.1
Systolic BP (mmHg)	136 (20)	109–179
Diastolic BP (mmHg)	84 (15)	57–117
Fasting insulin (pmol/l)	142 (105)	28–146
Fasting glucose (mmol/l)	5.5 (0.5)	4.7–6.3
GH (μg/l)	7.2 (10.0)	0.3–29.9
Total IGF1 (nmol/l)	79.7 (82.6)	28.0–140.0
IGFBP1 (μg/l)	1.27 (1.28)	0.00–4.20
IGFBP3 (mg/l)	7.0 (1.5)	4.6–8.7
IGF1 bioactivity (pmol/l)	589 (237)	218–1063
S-Klotho (ng/l)	2291 (2164)	488–6823
Ring size (mm)	22.0 (2.5)	17.5–24.5
Comorbidity (*n*)		
Secondary hypothyroidism	3	
Secondary hypogonadism	3	
Secondary adrenal failure	1	
Type 2 diabetes	3	
Hypertension	6	

BP, blood pressure.

**Table 2:** Individual age, total IGF1 levels, IGF1 bioactivity, and *Z*-scores of 15 subjects with active acromegaly.

**No.**	**Age** (years)	**Total IGF1** (nmol/l)	***Z*-score** (s.d.)	**IGF1 bioactivity** (pmol/l)	***Z*-score** (s.d.)
1	65.2	32.0	3.70	450	1.92
2	54.0	40.6	4.10	545	1.10
3	46.5	113.1	16.40	822	3.36
4	24.1	127.9	12.50	819	2.45
5	59.6	140.0	23.60	1063	6.28
6	49.5	43.3	4.20	361	0.41
7^a^	28.8	101.0	10.20	466	−0.07^b^
8	52.6	82.6	11.80	430	0.42
9	68.0	95.4	17.70	713	4.55
10^a^	69.2	78.9	14.10	434	1.11^b^
11	50.8	70.0	9.50	568	1.29
12	57.1	31.0	2.70	218	−1.55
13	38.2	104.4	13.20	770	2.56
14^a^	69.7	28.0	2.90	296	−0.59
15	59.1	107.0	17.30	828	4.11

a Type 2 diabetes; all three patients were using metformin.

b Using insulin.

**Table 3:** PCS of the SF-36, the MCS of the SF-36, the AcroQoL score, and PASQ in 15 patients with active acromegaly.

	**Baseline mean** (s.d.)	**Range**
SF-36		
PCS	45.6 (9.6)	28.6–58.7
MCS	43.1 (12.1)	24.6–55.6
AcroQol Questionnaire	67 (19)	32–97
Physical dimension	28 (9)	13–40
Psychological dimension	53 (10)	32–67
Appearance	22 (6)	13–32
Personal relations	30 (5)	19–35
PASQ	21.5 (13.3)	0.00–46.0

SF-36, Short-Form Health Survey-36 Questionnaire; PCS, physical component summaries; MCS, mental component summaries; AcroQol Questionnaire, Acromegaly Quality of Life Questionnaire; PASQ, Patient-Assessed Acromegaly Symptom Questionnaire.

**Table 4:** Age-adjusted relations in 15 subjects with active acromegaly between total IGF1 and IGF1 bioactivity, and the PCS of the SF-36, the MCS of the SF-36, the global AcroQol score, and the physical and psychological dimensions of the AcroQol Questionnaire and the PASQ.

**Baseline**	**Total IGF1**		**IGF1 bioactivity**	
*r*	*P* value	*r*	*P* value
SF-36				
PCS	−0.60	0.03*	−0.78	0.002*
MCS	−0.02	0.95	−0.18	0.55
AcroQol Questionnaire	−0.24	0.43	−0.39	0.19
Physical dimension	−0.37	0.19	−0.60	0.02*
Psychological dimension	−0.13	0.68	−0.19	0.53
Appearance	−0.23	0.44	−0.20	0.51
Personal relations	0.01	0.98	−0.16	0.61
PASQ	0.65	0.02*	0.57	0.05*

Statistically significant (*P<0.05). SF-36, Short-Form Health Survey-36 Questionnaire; PCS, physical component summaries; MCS, mental component summaries; AcroQol Questionnaire, Acromegaly Quality of Life Questionnaire; PASQ, Patient-Assessed Acromegaly Symptom Questionnaire.
